# Congenital Occlusion of the Postnasal Orifices, with Report of a Case

**Published:** 1913-09

**Authors:** Charles W. Richardson

**Affiliations:** Boston


### Congenital Occlusion of the Postnasal Orifices, With Report of a Case.

In July, 1912, a case came under my observation in which there was complete osseous obstruction of the postnasal orifices at as early a period in the life history of the patient as any observer has noted such a condition. In a search of the literature I have found only a few cases in which the obstruction was observed in infancy. While it is not possible to tabulate all the cases recorded in the literature, I judge that they do not exceed one hundred. The obstruction of the postnasal orifice may be membranous or osseous.

The former are usually found posterior to the nasal cavities in the nasopharyngeal cavity, but lie in contact with the postnasal orifices so as to completely obstruct them, while the latter are usually placed within the chamber within a millimeter or more from the free border of the posterior nasal orifice. To these two forms may be added congenital atresias, by which the bones entering into the formation of the postnasal orifice become united, thus more or less completely obstructing the postnasal orifices.

The child that came under my observation in July, 1912, had marked difficulty in breathing. The child struggled for air and the face became suffused and slightly cyanosed, the condition being relieved when the child began to cry. Whenever it ceased to cry there would be a recurrence of the difficult breathing. Examination demonstrated without doubt a deformity which was a complete obstruction of the postnasal orifices. By the end of the second week the child learned to maintain mouth breathing and also learned to feed in a short time, and has developed in a normal manner. The question is: When is the proper time to operate? The marked success with this case seemed to favor the expectant surgical policy in these cases.—Dr. Charles W. Richardson, Boston.

